# Development and validation of Medical Device Key Evidence Tool (‘MeDKET’): An evidence-based framework to explain success in selected European and US companies

**DOI:** 10.1371/journal.pone.0288126

**Published:** 2023-07-13

**Authors:** Stefania Manetti, Emanuele Lettieri, Melody Zhifang Ni

**Affiliations:** 1 Department of Management Engineering, Politecnico di Milano, Milano, Italy; 2 London In-Vitro Diagnostics (LIVD) Cooperative, Department of Surgery and Cancer, Imperial College London, London, United Kingdom; Universiteit Twente, NETHERLANDS

## Abstract

Innovating in Medical Device (MD) industry is challenging. This study aims to develop and validate an evidence-based framework that helps innovators of small and large enterprises (SEs and LEs) assess their readiness for successful MD development and deployment. We conducted a key-informant process (stage 1) where 25 international experts identified a list of emergent Health Technology Assessment (HTA) themes they believed were essential to company success. A sample of 22 European and US selected companies (13 SEs and 9 LEs) then reached a consensus on a list of key themes through a robust Delphi process (stage 2). Finally, we constructed (stage 3) and validated (stage 4) the checklist for SEs and LEs. The checklist for SEs and LEs included 21 and 15 items (i.e., fundamental Yes/No questions) with nine overlapping criteria for both SEs and LEs. In both groups, MD success was driven by three major item categories: (i) R&D assessment strategy; (ii) device-outcome measures; (iii) company profiling. Alongside the retrospective validation study, we collected 40 case studies on MDs (23 successes and 17 failures) across the selected enterprises. The retrospective validation provided the proportion of successful and failed case studies that met the ’MeDKET’ criteria. We discovered that early HTA plays a pivotal role in MD industry success with different implications based on enterprise size. This study is the first of its kind to provide a holistic picture of the perceived role of early-stage HTA in MD industry success.

## Introduction

The Research and Development (R&D) process in Medical Device (MD) industry is complex, expensive, and has plenty of pitfalls. This process is more multifaceted than the innovation continuum in other industries or sectors [1]. The unique complexity results from several factors: (i) the pressure from different stakeholders; (ii) the interface of many disciplines; (iii) the need for coordination between product development and clinical use through user involvement; (iv) the strong ethical implications; (v) the regulatory constraints [2, 3]. In this way, each R&D phase results sensitive to external constraints (i.e., clinical, legislative, social, ethical etc.), generating an interdependency among the different development phases than in other industries, including the pharmaceutical sector [4]. These dynamics create an accentuated need for feedback, continuous evidence generation and analysis alongside R&D using evaluation and audits [5].

Fewer than 6% of MDs reach the market each year after many years of development efforts and high costs sustained by companies, governments, and R&D investors, which could fund cost-ineffective technologies that never achieve implementation [4]. The development process in the MD industry may, therefore, require large amounts of time and resources but result in limited benefits for companies, governments, and -ultimately—society. The negative consequences also affect the whole society, which loses opportunities for healthcare improvements and maximising the return from the biomedical innovation efforts [5–7].

The specificities of the MD industry generate a particular case in the market access of new MDs due to the highly regulated aspect of the industry and the interdependencies of each new product with existing frameworks, products, and systems in clinical use [5, 8]. Indeed, the strictly regulated feature of the MD industry is similar to the automotive or nuclear industries, which are dynamic areas with permanent changes and a short lifetime of products [9]. Manufacturers have many regulatory constraints to satisfy for market approval, and these obligations continue even after selling the device, namely post-market surveillance and adverse event reporting [9]. Moreover, the barriers to commercialisation are related to technological (e.g., short life cycle), human-factor (e.g., user acceptability and adoption), and organisational challenges (e.g., existing alternatives) [4, 10, 11].

Technology producers should understand that the MD industry’s traditional definition of product success is evolving. New criteria and requirements for achieving success push relevant stakeholders involved in the R&D process (i.e., company, academia, and government) to assess innovative products as earlier as possible. The previous factors, combined with increasing healthcare budget constraints and reimbursement schemes, threaten the MD industry’s future, which is globally in accelerated and compulsory growth [12, 13].

Early-stage HTA is an emerging ’species’ of HTA in which technologies in development are evaluated to support healthcare decision-making from the initial idea up to phase III-like trials, anticipating market access and reimbursement [14, 15]. The rationale behind early-stage HTA is to inform internal investment decisions, select potential products or prototypes to take forward and avoid investments in new technologies that are less likely to succeed. In a later stage, these analyses can add value and efficiency to the road to reimbursement and coverage decisions. Early-stage HTA should make effective and affordable technology readily available to patients to maximise the benefits of biomedical R&D efforts for the public and society. The academic literature provides few reference guidelines for early-stage HTA [6, 16–19]. A critical factor for non-progression decisions at NICE Medical Technologies Evaluation Programme (MTEP) during its first three years (2010–2013) appears to be the little or no attention to an early economic evaluation of the new device by the manufacturer [20].

This exploratory study aims to develop and field-test an evidence-based framework to understand whether and to what extent early-stage HTA might influence the product success of small and large enterprises (SEs and LEs) from a company perspective. Incorporating early-stage HTA into R&D and company decision-making might be beneficial. However, evidence remains sparse on whether (and how) the MD industry conducts early-stage HTA. The implications of conducting early-stage HTA on future (post-market) product success must be made clear and straightforward. Our implicit aim is, therefore, to identify the drivers of MD industry success.

## Materials & methods

We applied a multi-method approach, which was successfully used in producing the ’POCKET’ toolkit [21] for developing an evidence-based framework for point-of-care testing devices.

**Fig 1:**
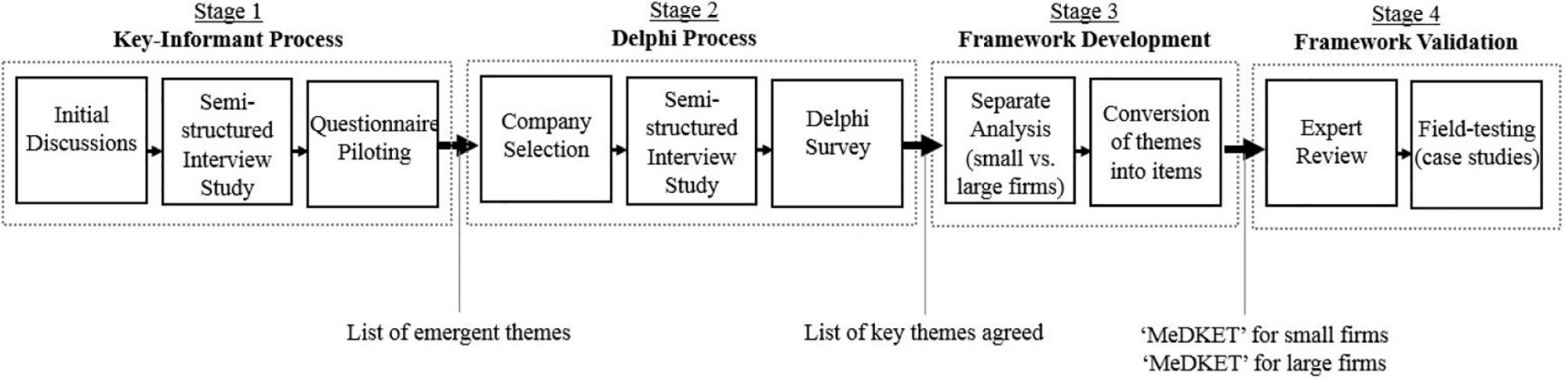
Study outline.

As outlined in Fig 1, this research encompassed four stages. We conducted a key-informant process (stage 1) where 25 international experts identified a list of emergent HTA themes pointed out as critical to company success. A sample of 22 European and US companies then reached a consensus on a list of key themes through a robust Delphi process (stage 2). In stage 3, we constructed the ’MEDKET’ checklist for SEs and LEs by defining and prioritising key themes using comments and ratings from stages 1 and 2. Finally, in stage 4, we field-tested ’MEDKET’ by collecting 40 case studies across the selected enterprises.

### Key-informant process

A three-step key-informant process was addressed to international experts and methodologists to identify a list of emergent themes that explained the MD company’s success.

#### Preliminary dialogues (Step 1)

Initial discussions at *Karolinska Institutet* involved senior academics in healthcare innovation management and informed the development of essential themes covered in the next step.

#### Semi-structured interview study (Step 2)

We conducted 25 semi-structured interviews by phone or in person with key informants (15 academics and ten industry professionals with experience-based or professional-based knowledge in the design, development, and deployment of MDs). The expert academics (2 from Italy and 13 from the UK) were identified by academic publishing output; some also worked as freelance consultants for the MD industry. The industry experts (6 from Italy and four from the UK) were well-known professionals working in spin-off companies based on our academic institutions. We performed a general thematic analysis of the interviews to extract emergent themes.

#### Questionnaire piloting (Step 3)

We translated the list of emergent themes, identified by the key informants, into a series of statements to form a survey questionnaire (Manetti et al., 2017). We piloted the questionnaire with the academic staff of the *Health Economics Research Centre* (*HERC*, *University of Oxford*) and re-piloted the revised version a month later. The aim was to make the easily understandable questions in case of no scientific background in biomedical technology innovation/early-stage HTA.

### Delphi process

A robust Delphi process with skilled professionals working in the MD industry aimed to reach a consensus on theme relevance.

#### Company selection (step 1)

We selected the Delphi participants at both company and professional levels. Inclusion criteria for SEs and LEs were as follows: (i) intense R&D process; (ii) high specialisation; (iii) international partners. We made every effort to include relevant experience or expertise existing outside Italy. We set a minimum of eight participants for each group (i.e., SEs and LEs) as a pragmatic sample size, given time and resources (Huddy et al., 2021).

Inclusion criteria for practitioners were as follows: (i) (at least) four years of experience within the company; (ii) a comprehensive view of R&D and market access (based on previous or current professional experience); (iii) accurate representation of the company needs and aims. We reached the leaders or CEOs of each firm by invitation included in their trade association newsletter (i.e., ASSOBIOMEDICA for Italy) or through direct contacts. After the approval of the CEO of each firm, we interviewed the CEO or the persons indicated by the CEO. We excluded six SEs after preliminary informal talks with the management team because the companies were not interested in this research and three LEs after their CEOs and other senior professionals were unavailable.

Results drove the final number of Delphi participants, as more interviews were carried out until no new themes emerged. Tables 1 and 2 show the sample for SEs (13 enterprises and 13 professionals) and for LEs (9 firms and 13 practitioners). The interviewees in the SE group gained 11.9±7.8 years of professional experience, whereas the practitioners in the LE group accumulated 13.2±8.7 years.

**Table 1:** Sample description of small enterprises.

Company	Interviewee	Role	Country
*Company J*	#1	Senior executive (including CEO)	Finland
*Company K*	#1	Managing director	Italy
*Company L*	#1	Senior executive (including CEO)	US
*Company M*	#1	Senior executive (including CEO)	Italy
*Company N*	#1	R&D officer	Italy
*Company O*	#1	R&D officer	Italy
*Company P*	#1	Senior executive (including CEO)	Austria
*Company Q*	#1	Market access officer	Finland
*Company R*	#1	Managing director	Finland
*Company S*	#1	Senior executive (including CEO)	Italy
*Company T*	#1	Senior executive (including CEO)	UK
*Company U*	#1	Senior executive (including CEO)	Italy
*Company V*	#1	Senior executive (including CEO)	Italy

**Table 2:** Sample description of large enterprises.

Company	Interviewee	Role	Country
*Company A*	#1	R&D officer	US
	#2	Global market access director	US
*Company B*	#1	Senior executive (including CEO)	US
	#2	Managing director	Italy
*Company C*	#1	Market access officer	Italy
	#2	Health economics manager	Italy
	#3	Market access officer	Italy
*Company D*	#1	Export manager	Switzerland
*Company E*	#1	R&D director	US
*Company F*	#1	Managing director	France
*Company G*	#1	Product line leader	Germany
*Company H*	#1	Health economics/reimbursement	Italy
*Company I*	#1	Senior executive (including CEO)	Switzerland

#### Semi-structured interview study (step 2)

The semi-structured interview study aimed to identify: (i) the degree of relevance of each HTA dimension in each R&D phase; (ii) starting time, approaches and perspectives used in early economic evaluation; (iii) barriers to market access and adoption of MDs; (iv) the costs and benefits of conducting early-stage HTA during R&D; (v) the perceived role of early-stage HTA in MD industry success.

We conducted 13 semi-structured interviews in the SE and 13 in the LE groups. Indeed, out of 9, 3 selected companies indicated more than one proper person. Out of 26, 15 interviews (12 in Italy and 3 in Finland) were conducted face-to-face by one interviewer (SM), whereas the remaining were via phone or "Skype". The interview structure resulted from the key-informant process (stage 1). It was further refined in conjunction with an expert in mixed research (MN) to allow the collection of both quantitative and qualitative data while consenting to flexibility and original suggestions. Interviews lasted for approximately 60 min, were digitally recorded, and then transcribed for analysis. The 12 interviews conducted in Italy were in Italian and translated into English. Two authors (SM and MN) co-analysed the data independently to minimise biases. Initially, interview data were coded based on predetermined themes; additional themes emerged and were coded during the interview analyses. In four cases, participants were contacted to clarify the interpretation of data. Data were analysed with NVivo V.10.1.1 software (QSR International, Melbourne, Victoria, Australia).

#### Delphi survey (step 3)

The Delphi questionnaire has been extensively described elsewhere [22]. The questionnaire statements were built on a five-point Likert scale (from 1 (strongly disagree) to 5 (strongly agree)) concerning theme relevance. Responders could also add free text comments alongside each statement if they felt it was needed to justify responses or suggest further new statements. Any new statements presented in free-text answers were iteratively added to the Delphi questionnaire. This iterative method allowed participants to reconsider their responses regarding the group results. This process was repeated until a consensus was reached. The consensus was set at ≥80% across statements that received ≥4 (agree or strongly agree).

The survey was administered online using a web method (Survey Monkey www.surveymonkey.com). In a few cases, the questionnaire was administered by phone; the score was assigned using the qualitative answers of the experts. Survey data were analysed using Stata 15 software for Windows (StataCorp. 2017. Stata Statistical Software: Release 15. College Station, TX: StataCorp LLC), and descriptive analyses were carried out (frequencies, median scores, range of scores).

### Framework development

The final stage aimed to construct the ’MEDKET’ checklist for SEs and LEs. The framework development was informed by the intermediate outputs from the previous stages, i.e., the list of emergent HTA themes (S1 Table) and the list of key themes agreed (S2 Table).

#### A separate analysis of small vs large firms (step 1)

We defined and prioritised key themes separately for small and large firms using comments and ratings from previous stages.

#### Conversion of themes into framework items (step 2)

First, we classified each prioritised theme into three major categories: (i) R&D processes; (ii) device outcome-measure; (iii) structure, which included structural peculiarities of the company and reference market segment. We organised the prioritised themes into two groups of relevance (i.e., primary and secondary) using comments and ratings from stages 1 and 2 combined with our interpretation. At last, we revised and edited the prioritised themes to ease the use of the checklist. We indifferently use the term’ item’ or ’driver’ to refer to the framework items.

### Ethics statement

The involvement of the Ethics Committee of Sant’Anna School of Advanced Studies was unnecessary, as the study recruited expert academics in HTA and MD developers involved in a qualitative study. There was no patient contact or involvement, and no access to patient data and medical records was necessary. The study was conducted by the EU General Data Protection Regulation EU-2016/679 (GDPR). Academic and industry experts were invited to participate through an information letter describing the study’s aims and methodology, and participants signed a written consensus. Data were stored on a protected server and collected anonymously.

## Results

### Definition of medical device success

We found out that SEs and LEs perceived success differently. Indeed, SEs perceived success as business continuity, whereas LEs identified success as large-scale utilisation and value experimented by patients and target users. The different perceptions of MD success led us to elaborate on two separate definitions based on enterprises’ size (Table 3).

**Table 3:** Definition of success for SEs and LEs.

Definition of success	Company group
The degree to which a company continues the device development and sells the product, generating revenue.	SE
The degree of market penetration.	LE
The degree to which a company improves patient outcomes and end-users experience using the medical device.	LE

The definitions of success as business continuity for SEs and market share for LEs involve measurable indicators that are relatively easy to obtain. The second definition of success for LEs, i.e., success perceived as achieving incremental value for patients and end-users, can be more challenging to quantify. "*We are very driven by patient value and whatever enables us to provide more value to the patients*. *That is a success*. *Patient outcomes are a success*. *No economic value*, *no simple technology development*. *If a surgeon finds value in using the device*. *That is a success*." CEO, large company]

### The ’MEDKET’ framework

Table 4 shows the ’MEDKET’ checklist combined for SEs and LEs, highlighting the overlaps and the differences between the two company groups.

**Table 4:** The ’MEDKET’ checklist for small and large enterprises.

Categories	Item	SEs	LEs
**R&D Processes**	**1**. Start from the identification of an unmet need.	Primary	Primary
	**2**. Incorporate the assessment of early usability into each phase of the R&D process.	Primary	Primary
	**3**. Incorporate the assessment of the early clinical dimension into each phase of the R&D process.	Primary	Not significant
	**4**. The involvement of stakeholders, especially target users (e.g., clinicians and patients), is part of each R&D phase.	Primary	Primary
	**5**. Invest the proper amount of time and resources to iteratively conduct early-stage HTA while acknowledging the existence of an "optimal" threshold between risks and benefits, beyond which the allocation of additional funding does not guarantee additional benefits ^a^	Not significant	Primary
	**6**. Adopt a ’prioritisation’ approach that maps the Technology Readiness Level (TRL) reached with the assessment dimension(s) that is(are) priority by assigning weights of relevance (e.g., pricing in TRL6 might have the maximum relevance) ^b^	Not significant	Primary
	**7**. Adopt a management review approach at the end of each R&D phase.	Not significant	Secondary
	**8**. Consider the design phase the ’upper time limit’ for an effective conduction of early-stage HTA.	Primary	Not significant
	**9**. Adoption of the user-centred design.	Primary	Not significant
	**10**. Employ the early-stage HTA results to ensure timely interruptions of unpromising products.	Not significant	Primary
	**11**. Timely conduct of early economic evaluations at least once before the design phase.	Primary	Not significant
	**12**. Exploit the results of early economic evaluations to build confidence in potential investors and raise funding for the subsequent development phases.	Secondary	Not significant
	**13**. Revise the analysis of users’ needs at the end of each R&D phase.	Secondary	Not significant
	**14**. Timely conduction of the (customer) market analysis at least once before the design phase.	Secondary	Not significant
**Structure**	**15**. Timely identification of funding needs to go to the market.	Primary	Not significant
	**16**. High standardisation and specialisation.	Primary	Primary
	**17**. Well-defined business model.	Primary	Secondary
	**18**. Being an incumbent in the reference market segment.	Primary	Primary
	**19**. Ensure organisational synergy between the key departments or units.	Not significant	Primary
	**20**. Look at new opportunities from emerging markets (e.g., developing countries).	Not significant	Secondary
	**21**. Being aware of the health economics concepts.	Secondary	Not significant
	**22**. Maintain active international distribution networks and relationships.	Secondary	Not significant
	**23**. Adequate Intellectual Property (IP) strategy.	Secondary	Not significant
**Device- outcome measures**	**24**. Offer a cost-effective device for the healthcare system and society.	Primary	Primary
	**25**. Offer value to patients and users beyond the device.	Primary	Primary
	**26**. Consider country-specific factors ^c^ that may affect the perceived value of the device.	Secondary	Primary
	**27**. Achievement of the (clinical) consensus to go to market.	Secondary	Not significant

^a^ Selected quotes can be found in S1 Appendix.

^b^ Selected quotes can be found in S2 Appendix.

^c^ Healthcare systems, funding and reimbursement mechanisms.

’MEDKET’ for SEs and LEs included 21 (13 primary and eight secondary) and 15 (12 primary and three secondary) items, with nine overlapping themes for both SEs and LEs. In both groups, success was driven by three major item categories: (i) R&D processes (e.g., starting time of assessment activities and identification of funding needs); (ii) device outcome measures (e.g., economic sustainability); (iii) structural peculiarities of the company (e.g., business model) and the specific market (e.g., being an incumbent in the reference market segment). Most ’MEDKET’ drivers belonged to the first item category (10 and 7 drivers for SEs and LEs, respectively).

### Field-testing of ’MEDKET’

The field-testing of the MEDKET checklist through 40 retrospective case studies collected from a selected sample of European and US small and large enterprises showed that primary and secondary framework items might drive MD success. Successful case studies had, on average, a more significant proportion of positive responses (i.e., Yes answers) on the ’MEDKET’ checklist compared with failed case studies. Similarities and differences between the successful and the failed case studies also emerged regarding the target MD (e.g., the purpose of use, risk class) and its innovation degree. Further information on the MDs assessed in the 40 case studies and successes/failures collected across case studies are detailed in S3 Table and S3 Appendix, respectively.

Table 5 presents how SEs performed on the ’MEDKET’ checklist by reporting the proportion of successful and failed case studies that met the related ’MEDKET’ criterion. The SE group gathered 12 MD successes and 8 MD failures. All the failures occurred before or very close to launching (i.e., TRL≥6). The starting time of early economic evaluations for the 20 case studies collected in the SE group is shown in S4 Appendix.

**Table 5:** Field-testing of ’MEDKET’ in small enterprises.

Items	SEs	Successes% (N = 12)°	Failures% (N = 8)^§^
Start from the identification of an unmet need.	Primary	67%	25%
Incorporate the assessment of early usability into each phase of the R&D process.	Primary	92%	13%
Incorporate the early clinical dimension assessment into each phase of the R&D process.	Primary	92%	13%
The involvement of stakeholders, especially target users (e.g., clinicians and patients), is part of each R&D phase.	Primary	92%	13%
Consider the design phase the ’upper time limit’ for an effective conduction of early HTA.	Primary	67%	13%
Adoption of the user-centred design.	Primary	100%	38%
Timely conduct of early economic evaluations at least once before the design phase.	Primary	84%	0%
Exploit the results of early economic evaluations to build confidence in potential investors and raise funding for the subsequent development phases.	Secondary	50%	0%
Revise the analysis of users’ needs at the end of each R&D phase.	Secondary	50%	0%
Timely conduction of the (customer) market analysis at least once before the design phase.	Secondary	84%	0%
Timely identification of funding needs to go to the market.	Primary	100%	0%
High standardisation and specialisation.	Primary	75%	38%
Well-defined business model.	Primary	67%	38%
Be an incumbent in the reference market segment.	Primary	67%	38%
Be aware of the health economics concepts.	Secondary	67%	38%
Maintain active international distribution networks and relationships.	Secondary	67%	25%
Adequate Intellectual Property (IP) strategy.	Secondary	67%	50%
Offer a cost-effective device for the healthcare system and society.	Primary	100%	0%
Offer value to patients and users beyond the device.	Primary	100%	0%
Consider country-specific factors that may affect the perceived value of the device.	Secondary	50%	0%
Achieve the (clinical) consensus to go to market.	Secondary	75%	0%

° Percentage of successful case studies meet the related criterion in the SE group.

^§^ Percentage of failed case studies meets the SE group’s related criterion.

Table 6 details the field-testing results by reporting the Percentage of successful and failed case studies that met the related ’MEDKET’ criterion. The LE group gathered 11 MD successes and 9 MD failures. Among the failed cases, 5 were post-market failures (i.e., TRL = 9), and four occurred before launch or very close to launching (i.e., TRL≥6).

**Table 6:** Field-testing of ’MEDKET’ in large enterprises.

Items	LEs	Successes% (N = 11)°	Failures% (N = 9)^§^
Start from the identification of an unmet need.	Primary	100%	56%
Incorporate the assessment of early usability into each phase of the R&D process.	Primary	100%	44%
The involvement of stakeholders, especially target users (e.g., clinicians and patients), is part of each R&D phase.	Primary	100%	44%
Invest the proper amount of time and resources to iteratively conduct early-stage HTA while acknowledging the existence of an "optimal" threshold between risks and benefits, beyond which the allocation of additional funding does not guarantee additional benefits.	Primary	82%	22%
Adopt a ’prioritisation’ approach that maps the TRL reached with the assessment dimension(s) that is(are) priority by assigning weights of relevance (e.g., pricing in TRL6 might have the maximum relevance).	Primary	82%	22%
Adopt a management review approach at the end of each R&D phase.	Secondary	64%	22%
Employ the early-stage HTA results to ensure timely interruptions of unpromising products.	Primary	82%	22%
High standardisation and specialisation.	Primary	100%	56%
Well-defined business model.	Secondary	100%	56%
Being an incumbent in the reference market segment.	Primary	100%	33%
Ensure organisational synergy between the key departments or units.	Primary	82%	44%
Look at new opportunities from emerging markets (e.g., developing countries).	Secondary	64%	22%
Offer a cost-effective device for the healthcare system and society.	Primary	100%	22%
Offer value to patients and users beyond the device.	Primary	100%	44%
Consider country-specific factors that may affect the perceived value of the device.	Primary	82%	44%

° Percentage of successful case studies meet the related criterion in the LE group.

^§^ Percentage of failed case studies meet the related criterion in the LE group.

## Discussions

In this exploratory study, we investigated the level of early-stage HTA incorporation into R&D and company decision-making using a four-stage mixed-method approach. The research culminated in a multiple case study that led us to obtain the external validation of the ’MEDKET’ framework. Moreover, we explored to what extent conducting early-stage HTA alongside biomedical R&D might drive future (post-market) product success by gathering retrospective case studies of successful and failed MDs from a selected sample of European and US small and large enterprises. The perceived role of early-stage HTA could have different implications on achieving success according to the enterprise size. Our study suggests original insights and reflections on the perceived role of early-stage HTA in achieving success for SEs and LEs.

Our study confirms evidence from the literature concerning the role of early-stage HTA in SEs. Small companies do not perform pre-market or early-stage analyses consistent with an early-stage HTA approach. This is not only due to the need for more resources and opportunities to conduct pre-market HTA but also to a specific knowledge-gap problem (i.e., skills, awareness, mentality). Many developers need to be made aware of health economics concepts, human-factor aspects, and market challenges, as they appear driven mainly by technological value and pioneer spirit. After several years of development efforts, SE developers realise they have to deal with the challenge of financial sustainability. However, last-minute adjustments are not possible at later stages when the device has already reached a high level of technology maturity. A "last-minute" approach to HTA may increase the risk of product failure and, for mono-product companies and start-ups, the risk of company failure. One company has failed during the conduction of the present study.

Our study sheds light on the perceived role of early-stage HTA in large companies to achieve post-market success. Our study provides evidence of the fact that LEs perform R&D analyses consistently with an early-stage HTA strategy, as conducting pre-market or early-stage HTA analyses can: (i) increase the overall R&D efficiency and efficacy; (ii) anticipate the interruption of devices in development that are likely to be unsuccessful in later R&D phases (*failing fast*, *failing cheap*); (iii) approach the R&D process more systematically while increasing the probability of future (post-market) product success. This evidence is well documented in the literature [7, 10, 14, 15, 23]. Third, investing the proper resources (i.e., funding, time, staff) to iteratively conduct early-stage HTA alongside R&D has been deemed necessary to reach post-market success. However, analysis quality, iterations’ number, and the choice of assessment dimensions represent a trade-off between investments and risks in large companies. During the Delphi study, experts acknowledged the existence of an ’optimum’ threshold of ’how’ to conduct early-stage HTA in each TRL, beyond which the allocation of additional resources can overcome the expected benefits for the company.

Moreover, LEs consider making early-stage HTA analyses more formalised and structured as unnecessary. The last point may explain the limited availability of case studies on early-stage HTA of innovative MDs in the published literature [24, 25]. Fourth, the perceived role of HTA in MD industry success changes between the pre-market and post-market phases. Early-stage HTA has been identified as a driver of future (post-market) product success. In contrast, the perceptions of mainstream or post-market HTA are controversial, also considering the lack of globally agreed rules on what and how constitutes evidence on new or novel MDs that will reach the market [3]. Mainstream HTA has been felt as a ’*necessary evil*’ whose negative results can affect market perceptions and MD success. However, the influence of positive impacts on MD reimbursement and adoption remains uncertain and unclear.

Our findings led us to draw a series of recommendations targeted to the crucial actors of the biomedical ecosystem (i.e., industry, academia, and governments).

Anticipating financial sustainability, health economics, and human factors alongside a biomedical technology’s lifecycle is essential for surviving the market. SEs should urgently adopt early-stage HTA to develop MDs more likely to meet the market’s expectations incorporating human-factor and financial requirements from the early stages of concept, design, and development. Furthermore, systematically integrating these requirements into the R&D process of innovative MDs might build more confidence in potential investors, ease searching for funding, and guarantee company survival and market continuum.

LEs should address the ’optimum’ threshold of ’how’ conducting early-stage HTA by adopting a ’prioritisation’ approach that maps the TRL reached by the product with the assessment dimension(s) that is(are) priority (e.g., pricing in TRL 6). Both SEs and LEs should increase and promote collaborations with academia, which can have a strategic role in conducting and disseminating early-stage HTA analyses. Finally, governments should provide comprehensive governance and leadership to define globally agreed rules on what and how constitutes reliable evidence for market approval and regulatory process of new or novel MDs to increase data sharing and public health transparency.

This research does present strengths and limitations. We highlight that the study was conducted using a company perspective. Thus, a mutual understanding of the implications associated with the perceived role of early-stage HTA as a driver of MD industry success needs to be further understood across other relevant stakeholders (i.e., policymakers, reimbursement bodies, research centres, and healthcare providers) with purposes of alignment. Another limitation concerns the adoption of a small sample size of participants that prevents us from drawing any conclusion regarding geographical variations. Whether (and how) the role of early-stage HTA in MD industry success varies considerably across countries is a critical consideration that needs further investigation. Finally, many study design limitations are typical to qualitative research, especially the Delphi process, which may lead to a compromise position rather than true consensus and a small sample size with unpredictable representation. Given the time and resources of this exploratory exercise, a certain degree of convenience sampling was required, as a random sampling approach was not feasible. Among the study strengths, we highlight (i) the use of a multi-method approach; (ii) a fruitful collaboration between academia and industry; (iii) the collection of multidimensional data on MD successes and failures by facing sparse and fragmented evidence characterising this emerging sector. In this sense, our study is the first to provide a holistic picture of the perceived role of early-stage HTAs in MD industry success.

## Conclusions

In this exploratory study, we investigated the level of early-stage HTA incorporation into MD R&D and company decision-making. Despite some limitations common to qualitative research, this study is the first to provide a holistic picture of the perceived role of early-stage HTAs in MD industry success. Our findings led us to draw relevant recommendations addressed to industry, governments, and academia to increase the efficacy and efficiency of the overall biomedical technology lifecycle.

## Supporting information

S1 Table List of emergent HTA themes. (DOCX)

S2 Table List of key themes agreed. (DOCX)

S3 Table Medical Devices assessed in the case studies. (DOCX)

S1 Appendix Verbatim quotation on the ‘MeDKET’ item #5. (DOCX)

S2 Appendix Verbatim quotation on the ‘MeDKET’ item #6. (DOCX)

S3 Appendix Successful and failed case studies. (DOCX)

S4 Appendix Starting time of early economic evaluation in SEs. (DOCX)
