# Supplementary material for: Development and validation of Medical Device Key Evidence Tool (‘MeDKET’): An evidence-based framework to explain success in selected European and US companies
Source: PLoS One. 2023 Jul 13;18(7):e0288126. doi: 10.1371/journal.pone.0288126 (PMC10343042; doi:10.1371/journal.pone.0288126)
Supplement: S1 Table — (DOCX) [file pone.0288126.s001.docx]

## Table S1 – List of emergent HTA themes

| **Emergent themes** |
| --- |
| The value of the early HTA strategy for innovative MDs from academia perspective. |
| The value of the early HTA strategy for innovative MDs from company perspective. |
| The areas or dimensions of the early HTA strategy for innovative MDs. |
| Starting time of early HTA activities in industry. |
| The priority of early HTA into R&D and company decision making. |
| Entity, types, and reasons of failures of innovative medical devices during the R&D. |
| Entity, types, and reasons of failures of innovative medical devices onto the market. |
| Company size as a critical issue for early HTA awareness. |
| Company size as a critical issue for early HTA conduction. |
| Potential economic benefits by applying rigorous methods of economic evaluations since early phases for companies. |
| Potential strategic benefits by applying rigorous methods of economic evaluations since early phases for companies. |
| Definition of medical device success from a company perspective. |
| Definition of medical device failure from a company perspective. |
| Reasons of successes from a company perspective. |
| Classification and definition of failures occurring in the different R&D phases. |
| How to effectively measure the time to market (or R&D) of an innovative medical device. |
| Issues and barriers to early HTA conduction in academia. |
| Issues and barriers to early HTA conduction in industry. |
| Issues around the market access and evidence required for widespread reimbursement from a company perspective. |
| Academia-Industry collaborations within early HTA. |
