# Supplementary material for: Development and validation of Medical Device Key Evidence Tool (‘MeDKET’): An evidence-based framework to explain success in selected European and US companies
Source: PLoS One. 2023 Jul 13;18(7):e0288126. doi: 10.1371/journal.pone.0288126 (PMC10343042; doi:10.1371/journal.pone.0288126)
Supplement: S2 Table — (DOCX) [file pone.0288126.s002.docx]

## Table S2 – List of key themes agreed

| **Key themes agreed** |
| --- |
| Being aware of the high entry barriers of European and US markets, which are extremely regulated and consolidated. |
| Being aware of the health economics concepts. |
| Maintain the company’s market segment (i.e. device area, business unit etc.). |
| A common reason for discontinuing projects in later R&D phases is the availability of an updating technology version onto the market. |
| Being aware of the proper economic evaluation methodology according to the perspective of analysis (i.e. internal perspective vs outside company perspective). |
| The lack of funding to complete the development and/or to go-to market is a relevant reason of pre-market failures proportionally to the size of company. |
| EU or national research projects are attractive sources of funding for small companies, especially those born as spin-offs or close to academia. |
| Have a robust and clear business model. |
| High standardization and specialization. |
| Maintain adequate distribution networks and international relationships. |
| Proper management of the co-development projects and related partners. |
| An underestimation of market dynamics and competitors, whose analysis should be undertaken in early R&D phases, is a common reason of failures close to launch or after launch proportionally to the size of company. |
| Need for the (clinical) consensus to go-to market proportionally to the size and maturity of company. |
| Medical device success may be affected by country-specific determinants (e.g. reimbursement mechanisms, health-care system etc.). |
| Start from the identification of (unmet) user needs in the research phase. |
| Usability (or user needs) is the most relevant dimension of pre-market analysis influencing each R&D phase. |
| Involve all relevant stakeholders (i.e. customers, consumers, payers, clinicians, consultants etc.) in each R&D phase. |
| Identify different levels of final users (e.g. customer, consumer, payer etc.) and evaluate their needs separately. |
| Consider any dimension of pre-market analysis in any R&D phase by changing the degree of relevance or impact according to the phase. |
| Acknowledge the existence of an ‘upper time limit’ for an effective application of early HTA. |
| Consider the design phase as the ‘upper time limit’ for an effective application of early HTA. |
| Acknowledge the existence of an optimum level of early HTA intensity beyond which risks can overcome benefits. |
| Adoption of management review-phase approach to evaluate processes and products at each progression to the next R&D phase. |
| Well-timed identification of funding needs to continue the development and/or go-to market. |
| Consideration of an ‘outside-in’ perspective looking at new opportunities from emerging markets (e.g. developing countries). |
| Adoption of the user centred design. |
| Adequate Intellectual Property (IP) strategy. |
| Failures can be classified according to the R&D phase: (i) failures occurring in the early R&D phases; (ii) failures close to launch or last minute; (iii) failure after launch. The extent of damage increases proportionally with the phase. |
| When doing an in-depth R&D, the first phase (i.e. research phase) is similar to academic research, and the failures at stake are ‘intrinsic risks’ of scientific research. |
| The reasons of failures occurring in the first R&D phases (i.e. research and concept phases) are mostly technical/design (e.g. reliability issues). |
| The highest failures are observed before the decision to advance towards the formal development process. |
| Difficulty in reporting failure rates characterizing the early R&D phases. |
| A very small amount of money is usually spent before the decision to advance towards the formal development process. |
| Failure rates close to launch or after launch are usually smaller than those characterizing previous R&D stage. |
| The lack of (organizational) synergy among key departments or units is a common reason of discontinuing projects in later R&D phases. |
| The achievement of a balance between benefits and risks in conducting early HTA changes according to the resource constraints, company confidence and expectations in a given project. |
| Late HTA is usually a negative risk. Indeed, negative results certainly affect the future device success; however, positive results does not necessarily affect funding and reimbursement decisions. |
| The improvement of clinical outcomes is very important, however it is not enough alone. |
| The lack of device cost-effectiveness, especially economic sustainability, is a key reason of failures after launch. |
| Unsatisfied user expectations is a key reason of failures after launch. |
