# Supplementary material for: Development and validation of Medical Device Key Evidence Tool (‘MeDKET’): An evidence-based framework to explain success in selected European and US companies
Source: PLoS One. 2023 Jul 13;18(7):e0288126. doi: 10.1371/journal.pone.0288126 (PMC10343042; doi:10.1371/journal.pone.0288126)
Supplement: S3 Table — (DOCX) [file pone.0288126.s003.docx]

## Table S5 – Medical Devices assessed in the case studies

| **Area** | **Possible value** |  |  |  |  |
| --- | --- | --- | --- | --- | --- |
| **Technology purpose** | Diagnostic | Surgery | Therapeutic | Rehabilitation | Monitoring |
| **Risk category** | I | II | III |  |  |
| **Invasiveness** | Implantable | Invasive in orifices | Invasive surgical | Non-invasive |  |
| **User interaction** | Yes | No |  |  |  |
| **Diagnostic type** | Near or at the point of care | Away |  |  |  |
| **Contact type** | No contact | Short term | Temporary | Long-term |  |
| **Active device** | Yes | No |  |  |  |
| **Operator sensitive** | Yes | No |  |  |  |
| **Persuasive device** | Yes | No |  |  |  |
| **Target organs** | Central/Cardio system | Other |  |  |  |

| **Outcome ^a^** | **Company size ^b^** | **Time ^c^** | **Inn**  **Degree ^d^** | **Year** | **R&D years** | **Risk class** | **Device purpose** | **Invasiveness** | **User interaction** | **Active device** | **Operator sensitive** | **Persuasive device** | **Target organs** | **Type diagnostics** |
| --- | --- | --- | --- | --- | --- | --- | --- | --- | --- | --- | --- | --- | --- | --- |
| F | **SE** | P | I | 2009 | <3 | IIA | Surgery | Non-invasive | No | Yes | Yes | No |  |  |
| F | **SE** | P | I | 2016 | <3 | IIA | Rehabilitation | Non-invasive | Yes | Yes | Yes | No | Other |  |
| F | **SE** | P | I | 2005 | >6 | I | Rehabilitation | Non-invasive | Yes | No | No | No | Other |  |
| F | **SE** | P | I | 2015 | 3 to 5 | IIA | Rehabilitation | Non-invasive | Yes | Yes | No | Yes | Other |  |
| F | **SE** | P | I | 2007 | >6 | IIA | Rehabilitation | Non-invasive | Yes | Yes | No | No | Other |  |
| F | **SE** | P | I | 2014 | <3 | I | Diagnostic | Non-invasive | No | No | No | No |  |  |
| F | **LE** | M | I | 2012 | 3 to 5 | IIA | Non-surgery therapeutic | Invasive in orifices | Yes | No | No | Yes | Other |  |
| F | **LE** | P | I | 2016 | >6 | IIA | Diagnostic | Non-invasive | No | No | Yes | No |  | Near or at the point of care |
| F | **LE** | P | I | 2015 | >6 | IIA | Diagnostic | Non-invasive | No | Yes | Yes | No |  | Near or at the point of care |
| F | **LE** | P | I | 2013 | <3 | IIB | Monitoring | Invasive in orifices | Yes | Yes | No | No | Other |  |
| F | **LE** | M | I | 2016 | 3 to 5 | III | Non-surgery therapeutic | Implantable | Yes | No | No | No | SNC/CCC |  |
| F | **SE** | P | I | 2012 | >6 | IIB | Monitoring | Non-invasive | No | Yes | No | No | SNC/CCC |  |
| F | **LE** | P | I | 2014 | 3 to 5 | III | Non-surgery therapeutic | Implantable | Yes | Yes | No | No | SNC/CCC |  |
| F | **SE** | P | I | 2014 | <3 | I | Diagnostic | Non-invasive | No | No | No | No |  | Near or at the point of care |
| F | **LE** | M | I | 2012 | 3 to 5 | IIA | Non-surgery therapeutic | Invasive in orifices | Yes | Yes | No | No |  |  |
| F | **LE** | M | I | 2017 | <3 | III | Non-surgery therapeutic | Implantable | Yes | Yes | No | No | SNC/CCC |  |
| F | **LE** | M | I | 2014 | 3 to 5 | III | Diagnostic | Invasive | Yes | Yes | Yes | No | SNC/CCC | Near or at the point of care |
| S | **SE** | M | I | 2009 | <3 | IIA | Non-surgery therapeutic | Invasive in orifices | Yes | Yes | No | No | Other |  |
| S | **SE** | M | I | 2018 | >6 | IIA | Rehabilitation | Invasive in orifices | Yes | Yes | No | No | Other |  |
| S | **SE** | M | R | 2018 | 3 to 5 | IIA | Rehabilitation | Invasive in orifices | Yes | Yes | No | No | Other |  |
| S | **SE** | M | R | 2015 | >6 | IIA | Rehabilitation | Non-invasive | Yes | Yes | No | No | Other |  |
| S | **SE** | M | R | 2017 | >6 | IIA | Rehabilitation | Non-invasive | Yes | Yes | No | No | Other |  |
| S | **SE** | M | R | 2015 | >6 | IIA | Monitoring | Non-invasive | Yes | Yes | No | No | Other |  |
| S | **SE** | M | I | 2015 | <3 | III | Non-surgery therapeutic | Implantable | Yes | Yes | No | No | SNC/CCC |  |
| S | **LE** | M | I | 2015 | 3 to 5 | III | Surgery | Invasive surgical | Yes | Yes | Yes | No | SNC/CCC |  |
| S | **LE** | M | I | 2013 | <3 | IIA | Non-surgery therapeutic | Invasive in orifices | Yes | No | No | Yes | Other |  |
| S | **LE** | M | I | 2015 | >6 | IIA | Diagnostic | Non-invasive | No | Yes | Yes | No |  | Near or at the point of care |
| S | **LE** | M | I | 2013 | >6 | III | Non-surgery therapeutic | Implantable | Yes | Yes | No | No | SNC/CCC |  |
| S | **LE** | M | I | 2015 | 3 to 5 | III | Non-surgery therapeutic | Implantable | Yes | No | No | No | SNC/CCC |  |
| S | **LE** | M | R | 2014 | >6 | IIA | Surgery | Invasive surgical | Yes | Yes | Yes | No | SNC/CCC |  |
| S | **LE** | M | R | 2014 | >6 | III | Non-surgery therapeutic | Implantable | Yes | Yes | No | No | SNC/CCC |  |
| S | **LE** | M | I | 2015 | >6 | III | Non-surgery therapeutic | Implantable | Yes | Yes | No | No | SNC/CCC |  |
| S | **SE** | M | I | 2014 | >6 | IIB | Diagnostic | Non-invasive | Yes | Yes | No | No | SNC/CCC | Near or at the point of care |
| S | **SE** | M | I | 2017 | >6 | I | Diagnostic | Non-invasive | No | No | No | No |  | Near or at the point of care |
| S | **SE** | M | I | 2014 | >6 | IIA | Diagnostic | Non-invasive | No | Yes | No | No |  | Near or at the point of care |
| S | **SE** | M | I | 2018 | >6 | IIA | Diagnostic | Non-invasive | Yes | Yes | No | No |  | Near or at the point of care |
| S | **SE** | M | I | 2018 | >6 | IIA | Diagnostic | Non-invasive | No | No | No | No |  | Near or at the point of care |
| S | **LE** | M | R | 2018 | >6 | IIA | Rehabilitation | Non-invasive | Yes | Yes | Yes | No | Other |  |
| S | **LE** | M | I | 2017 | >6 | III | Non-surgery therapeutic | Implantable | Yes | Yes | No | No | SNC/CCC |  |
| S | **LE** | M | I | 2014 | >6 | IIA | Surgery | Invasive surgical | Yes | Yes | Yes | No | SNC/CCC |  |

^a^ Possible values: F (Failure) or S (Success).

^b^ Possible values: SE (Small Enterprises) or LE (Large Enterprises).

^c^ Possible values: P (Pre-market) or M (Market) or NA (Not Applicable).

^d^ Possible values: I (Incremental) or R (Radical).
