# Supplementary material for: Development and validation of Medical Device Key Evidence Tool (‘MeDKET’): An evidence-based framework to explain success in selected European and US companies
Source: PLoS One. 2023 Jul 13;18(7):e0288126. doi: 10.1371/journal.pone.0288126 (PMC10343042; doi:10.1371/journal.pone.0288126)
Supplement: S1 Appendix — (DOCX) [file pone.0288126.s004.docx]

## Appendix S3 – Verbatim quotation on the ‘MeDKET’ item #5

**2nd order codes**: ‘Optimum’ threshold to the iterative application of early-stage HTA

**Selected quote on 1st order codes** “*Our approach to early-stage HTA analyses is iterative in the following acceptation. We conduct an unstructured and simple early-stage HTA analysis in the first stages of product development. We iteratively re-visited these analyses as further evidence becomes available and the product development seems promising. However, only at the mature point of a “later design” or “testing phase” (5≤TRL≤6), when we are close to achieving a prototype, these kinds of ‘personal’ analyses become more complex early-stage HTA analyses. We have no interest in making early-stage HTA analyses more complete or structured*” [CEO, large company].
