# Supplementary material for: Development and validation of Medical Device Key Evidence Tool (‘MeDKET’): An evidence-based framework to explain success in selected European and US companies
Source: PLoS One. 2023 Jul 13;18(7):e0288126. doi: 10.1371/journal.pone.0288126 (PMC10343042; doi:10.1371/journal.pone.0288126)
Supplement: S3 Appendix — (DOCX) [file pone.0288126.s006.docx]

## Appendix S6 – Successful and Failed case-studies

## For both SEs and LEs, MD success happens at the end of the development pathway, after launch, being always a post-market success.

## On the contrary, failures happen in the market and at any time alongside the R&D journey. We found out that in LEs, on average, the category of failures happening in early TRLs (<4-5) is close to 30%. and reasons for failures are mostly technical. Developers have innovative ideas and believe in the customer value of this idea, but technical delivery may be unfeasible. The second category of failures is less than 30% and happens very close to launching (TRLs≥7) with MDs that do not meet the company expectations (e.g., unpredictable changes in market perceptions and organisational discrepancies among the key units). Finally, the third category involves failures in the market, its percentage is minor than 15%, and it can affect the company’s image towards investors and customers. Most post-market failures are due to economic issues of the device (e.g., economic unsustainability and lack of cost-effectiveness), as well as the replacement of the product by newer generations.
