# Supplementary material for: Development and validation of Medical Device Key Evidence Tool (‘MeDKET’): An evidence-based framework to explain success in selected European and US companies
Source: PLoS One. 2023 Jul 13;18(7):e0288126. doi: 10.1371/journal.pone.0288126 (PMC10343042; doi:10.1371/journal.pone.0288126)
Supplement: S4 Appendix — (DOCX) [file pone.0288126.s007.docx]

## Appendix S7 – Starting time of early economic evaluation in SEs

The conduction of well-timed early economic evaluations is a driver of MD success; however, conducting early economic evaluations in later stages might be a driver of product failure. We identified the design phase as the ‘upper time limit’ for effectively conducting early economic evaluations and, more generally, early-stage HTA activities.
